# Incidental findings are frequent in shoulder CT and MRI scans and increase with age

**DOI:** 10.1016/j.jor.2024.05.024

**Published:** 2024-05-27

**Authors:** Mario Pasurka, Adrian Statescu, Philipp von Knebel Doeberitz, Joshua Kubach, Franz Dally, Sascha Gravius, Marcel Betsch

**Affiliations:** a Department of Trauma Surgery and Orthopaedics, University Hospital Erlangen, Friedrich-Alexander-University Erlangen-Nuremberg, 91054, Erlangen, Germany; b Department of Orthopaedic and Trauma Surgery, University Medical Center Mannheim, Medical Faculty Mannheim of the University of Heidelberg, 68167, Mannheim, Germany; c Department of Radiology and Nuclear Medicine, University Medical Center Mannheim, Medical Faculty Mannheim of the University of Heidelberg, 68167, Mannheim, Germany

**Keywords:** Shoulder imaging, Incidental finding, Incidentaloma, CT, MRI

## Abstract

**Objectives:**

CT and MRI scans of the shoulder can lead to the identification of incidental findings (IF), which can have a major impact on the further treatment of the patient. The aim of this retrospective study was to record the prevalence of IF, incidentalomas (IT) and malignant IT for CT and MRI examinations of the shoulder and to investigate the effect of patient characteristics on the statistical occurrence of IF, IT and malignant IT.

**Materials and methods:**

A total of 903 shoulder examinations (415 CT, 488 MRI) were retrospectively analyzed for the presence of IF, subsequently categorized (harmless IF, IT requiring clarification, malignant IT) and analyzed regarding patient characteristics. The statistical analysis was carried out using independent t- and chi-square tests. A significance level of p < 0.05 was set.

**Results:**

Among the 903 patients evaluated (436 female, 467 male), 153 (16.9%) patients experienced IF (harmless IF: 101 (11.2%) patients, IT: 94 (10.4%), malignant IT: 4 (0.4%). The average age of the patients without IF and IT was significantly lower compared to the patients with IF and IT (p < 0.001). While IF occurred in 31.1% of the CT, IF was only detected in 4.9% of the MRI (p < 0.001).

**Conclusion:**

IF have a high prevalence (16.9%), especially in CT examinations of the shoulder, which increases with age. The exact detection and initiation of appropriate therapy is of great clinical importance, as early detection of life-threatening diseases enables more effective treatment and a potential gain in health and lifespan.

## 1 Introduction

In addition to the medical history and physical examination, the diagnosis and treatment of shoulder diseases relies heavily on radiological imaging. The standard imaging technique for assessing pathologies such as fractures, dislocations, bony injuries, soft tissue calcifications and joint deformities is conventional x-ray. For the diagnosis of more complex pathologies, such as rotator cuff tears, tumorous and inflammatory diseases, but also for the preoperative planning of total shoulder replacements, computed tomography (CT) and magnetic resonance imaging (MRI) are currently used. As both technologies continue to evolve, the number of imaging examinations will continue to rise in the near future.^1^ With special positioning, diagnosis-specific sequencing such as fat suppression, spin-echo and proton-density techniques, and higher power magnets (3.0 T), MRI examinations allow for an unprecedented level of soft tissue imaging.^2^ MRI and CT scans also contribute to the pre- and intraoperative planning process, as 3D planning software for shoulder arthroplasty are utilized for aiding in the intraoperative determination of the glenoid component position.^3^^,^^4^ When evaluating these high-resolution diagnostic studies, a high number of incidental findings (IF) can be expected, because the field of view includes also other structures like the thoracic cavity and lungs due to their morphologic relation.^4^^,^^5^ Possible IF include pneumonia, bronchial carcinoma, metastatic lymph nodes, pulmonary hypertension, cystic and pulmonary fibrosis.^6^ Especially malignant findings illustrate the importance of vigilant reporting and image reviewal by both the radiology and surgical teams. Although most findings including lung nodules are benign,^7^ in case of cancerous pathologies an appropriate triage and workup is crucial. Incidental detection of such pathologies, at an early stage, can lead to more effective treatment with a potential gain in clinical outcome and lifespan.^8^ While previous studies determined the prevalence of IF in shoulder CT scans, this is the first study including MRI findings and comparing these two modalities regarding IF findings. Purpose of this retrospective work was to investigate the prevalence of IF, incidentalomas (IT) and malignant IT for CT and MRI examinations of the shoulder. Additionally, the effect of various patient characteristics including age, weight, height and gender as well as the influence of contrast agent administration on the statistical occurrence of IF, IT and malignant IT was evaluated.

## 2 Materials and methods

### 2.1 Study design and ethics approval

The present study is a retrospective, monocentric study at a Department of Orthopedics at the University Hospital XXX. A positive vote from the local Ethics Committee has been received (sign: 2021-854).

### 2.2 Data collection

The patient data from the CT and MRI examinations were provided by the Department of Radiology of the University Hospital XXX. The radiological findings and reports were analyzed and completed by evaluating the digital patient files. The patient data was recorded in pseudonymized form in a database and graphically displayed using Microsoft® Excel (Microsoft Corporation, Redmond, USA).

### 2.3 Patient collective

**Fig. 1:**
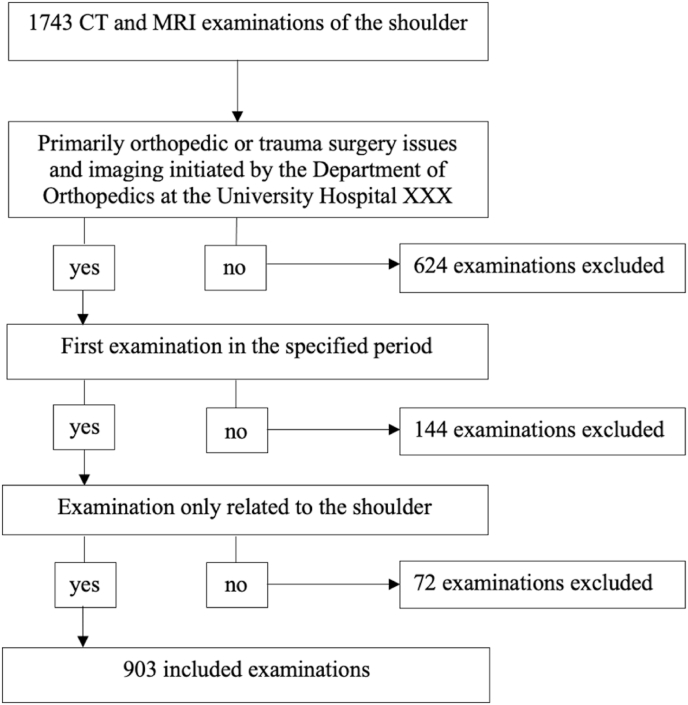
Flow chart of the included examinations.

1743 radiological studies of patients who received a CT or MRI examination of the shoulder between 2011 and 2021 were primarily included. Among these, all examinations of the shoulder with a primary orthopedic or trauma related question and which were indicated by the Department of Orthopedics (n = 1119) were included in this study. In addition, studies were excluded whose examination field of view was too small to show relevant other parts of the body besides the shoulder. For each patient, only the first examination in the specified evaluation period was evaluated. With this, 144 repeated examinations were excluded and each examination corresponds to a single patient. The entire patient population therefore comprised of 975 patients, of which 472 (48.4%) patients received a CT examination and 503 (51.6%) patients received an MRI examination. The entire study population (n = 975) was further evaluated regarding the orthopedic indication of each examination and a distinction was made as to whether it was only related to the shoulder or whether it also included other anatomical regions. Further regions included e.g. Skull, neck, thorax, abdomen and pelvis. Only the examinations that exclusively examined the shoulder region were included (n = 903) (Fig. 1).

### 2.4 Recording and classification of incidental findings

The radiological findings of each included examination were examined for the identification of IF and were included in the database. Only IF detected outside the shoulder which were previously unknown were considered. As mentioned before, each examination could yield multiple IF. Each IF was assigned to one of several possible categories based on its clinical relevance. IF, which had only little clinical relevance, were not pursued further, but were directly categorized as harmless IF^9^ (Table 1).

**Table 1:** Harmless IF of the respective anatomic region (IF: incidental finding).

Anatomic region	Harmless IF
**Head**	frontal hyperostosis, swelling or obstruction of the paranasal sinuses, osteoarthritis of the temporomandibular joint
**Neck**	arteriosclerosis, carotid elongation, carotid stenosis, hypoplastic vertebral artery, edema
**Spine and Skeletal**	degenerative changes, previous rib fracture, bone hemangioma, osteopenia, osteoporosis, rib sclerosis
**Thorax**	arteriosclerosis, fibroadenoma, gynecomastia, calcified lymph nodes, edema, lipomatosis, residual thymic tissue o Aorta: Aortic elongation, aortic kinking, aortic sclerosis o Axilla: hematoma, non-specific lymph nodes o Vascular anomalies: Artery lusoria, collateral circulation o Heart: cardiac hypertrophy, cardiac valve sclerosis, coronary sclerosis o Lung: atelectasis, dystelectasis, pleural calcification, lobe venae azygos
**Abdomen**	arteriosclerosis, elevated diaphragm, stenosis of the celiac trunk o Gallbladder: cholelithiasis o Liver: liver cyst o Stomach: Hiatal hernia o Spleen: Accessory spleen, splenomegaly o Kidney: urinary stasis, nephrolithiasis, renal atrophy

The categorization as harmless IF is consistent with the classification of Lumbreras et al.,^9^ whereby the findings referred as benign IF fall into the categories of findings with low and moderate clinical relevance. Thus, all other IF recorded in the radiological report were classified into the IT category. These cases were compared with the patient file to determine what clinical relevance resulted. If there was no clinical relevance, it was classified into the category of harmless IF. If it turned out to be malignant, it was recorded as malignant IT. Therefore, the description of the findings corresponds to the description in the radiological reporting and the confirmed relevance is reflected in the listing of the findings in the corresponding IF category (Table 2).

**Table 2:** Categorization of Incidental findings (IF: Incidental finding, IT: Incidentaloma).

IF Categories	Description
**Harmless IF**	IF that most likely had no direct or little clinical relevance and required no further action
**IT**	IF that required further clarification or activity
**Malignant IT**	IF which is confirmed to be malignant by further investigation received disease value

A step-by-step analysis of the patients and their characteristics was carried out based on the relevance of the findings in the three different IF categories. First, the patients who had IF with the highest relevance or disease value were considered as malignant IT, subsequently patients with IT and finally patients with IF. This made it possible to achieve a more differentiated determination of the prevalence for different scenarios according to IF relevance and scope of examination.

### 2.5 Recording of additional clinical parameters

In addition to the IF, other patient and examination related data were recorded and evaluated. This includes data such as age, height and weight. It should be noted that information about height was only recorded for 40.5% (n = 395) and weight for only 47.2% (n = 460) of the total 975 patients, due to lack of data in patient files. Therefore, all information on height and weight refers only to these patients. In addition, two age groups were formed, with a distinction being made between adults (18–64 years) and seniors (65 years and older). This classification was based on the definitions of age groups as found in the Medical Subject Headings (MeSH) Thesaurus of the United States National Library of Medicine and used for indexing articles in the MEDLINE database.^10^ In addition, data such as gender, contrast agent administration and examination modality were also analyzed. The laterality of the extremity was also recorded during the examination.

### 2.6 Evaluation and statistical analysis

The evaluation and statistical analysis of the data using independent t- and chi-square tests was carried out with the program IBM SPSS® Statistics (IBM Germany, Ehningen, Germany). The Fisher-Boschloo exact test was calculated using the NC State University website (Department of Statistics NC State University, 1996). P-values of <0.05 were regarded as statistically significant.

## 3 Results

### 3.1 Demographic data of the patient population

**Fig. 2:**
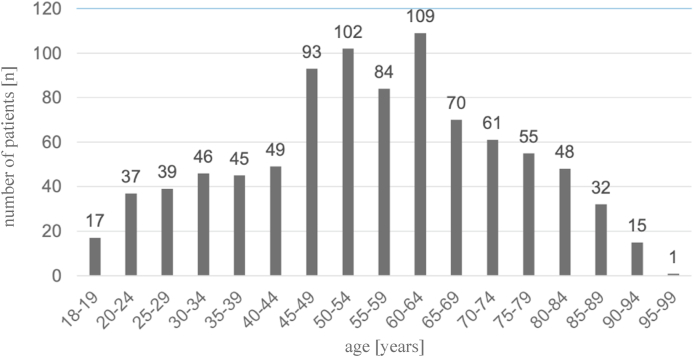
Distribution of age among included patients.

A total of 903 patients were included in the study (436 (48.3%) females, 467 (51.7%) males, age: 55.41 ± 18.08 years). 621 (68.8%) patients belonged to the age group between 18 and 64 years and 282 (31.2%) to the age group of at least 65 years. In the age group of 18–64 years, the average age was 46.05 ± 12.95 years, in the group of at least 65 years average age was 76.03 ± 7.67 years. When the patients were separated according to the examination modality, the average age of the CT population was 62.78 ± 18.71 years, whereas that of the MRI population was 49.14 ± 14.91 years. Height was measured for 364 (40.3%) patients and ranged from 140 cm to 198 cm (170.36 ± 10.07 cm). Weight was reported for 420 (46.5%) patients and ranged from 30 kg to 150 kg (81.05 ± 19.82 kg) (Table 3, Fig. 2).

**Table 3:** Demographic data of patient population (SD = standard deviation).

	n	proportion of patients [%|	average	SD
**Age [years]**	903	100	55.41	18.08
**Age Group 18**–**64 [years]**	621	68.8	46.05	12.95
**Age Group >65 [years]**	282	31.2	76.03	7.67
**Height [cm]**	364	40.3	170.36	10.07
**Weight [kg]**	420	46.5	81.05	19.82

### 3.2 Incidental findings

**Fig. 3:**
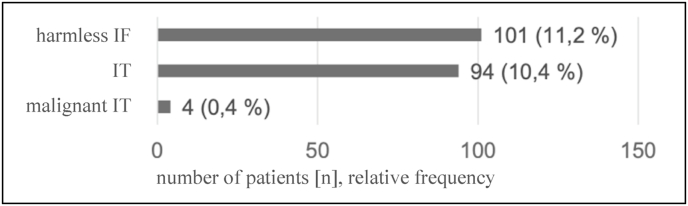
Occurrence of patients with different IF (patients can occur multiple times).

Among the 903 patients evaluated, IF occurred in 153 (16.9%) patients (harmless IF: 101 (11.2%), IT: 94 (10.4%), malignant IT: 4 (0.4%)). A total of 321 IFs (191 harmless IF, 117 IT and 13 malignant) occurred among these 153 patients, with each of these patients having between one to ten IFs (mean = 2.10) (Fig. 3).

### 3.3 Harmless incidental findings

191 harmless IF occurred in 101 patients. The most common benign IF were degenerative changes of the thoracic spine, which occurred in 32 (3.5%) patients, followed by aortic sclerosis in 24 (2.7%) and other degenerative changes of the skeleton in 21 (2.3%) patients. In contrast to degenerative changes in the thoracic spine, the latter related to the entire skeleton.

### 3.4 Incidentalomas

117 IT in 94 patients were revealed during the evaluation. The most common IT were enlarged axillary lymph nodes in 23 (2.5%) patients, followed by thyroid goiter in 22 (2.4%) patients and pulmonary nodules in 18 (2.0%) patients.

### 3.5 Malignant incidentalomas

During the investigation period, 13 IT turned out to be confirmed malignant in four patients. The most common malignant IT were osteolysis of the cervical spine and the skeleton, as well as pulmonary nodules, each occurring twice (0.2%).

### 3.6 Analysis of incidental findings

Patients without IF were statistically significant younger (p < 0.001) and heavier (p = 0.002) than patients with IF. No statistical difference was found regarding height (p = 0.074). While the average age of patients without IT was 12.52 years lower (p < 0.001) and patients without IT weighing an average of 7.11 kg more (p = 0.014) than patient with IT, there was no significant difference regarding height (p = 0.180). No statistically significant differences regarding age, weight and height were obtained regarding the appearance of malignant IT (Table 4).

**Table 4:** Comparison of IF findings regarding age, weight and height (IF: incidental finding, IT: incidentaloma, MIT: malignant incidentaloma).

	IF	**∅** IF	p (IF vs. **∅** IF)	IT	**∅** IT	**p** (IT vs. **∅** IT)	MIT	**∅** MIT	p (MIT vs. **∅** MIT)
**Age [years]**	66.48 ±19.29	53.15 ±16.97	**<0.001**	66.63 ±17.70	54.11 ±17.68	**<0.001**	71.50 ±11.21	55.34 ±18.08	0.074
**Weight [kg]**	75.03 ±18.11	82.46 ±19.97	**0.002**	74.85 ±16.78	81.96 ±20.09	**0.014**	70.33 ±13.05	81.12 ±19.85	0.348
**Height [cm]**	168.38 ±9.43	170.81 ±10.16	0.074	168.45 ±8.55	170.63 ±10.24	0.180	168.67 ±5.51	170.38 ±10.10	0.770

Analysis of gender remained without significant differences (IF: 19.0% women vs. 15.0% men, p = 0.105; IT: 12.2% women vs. 8.8% men, p = 0.097; malignant IT: women 0.5% vs. 0.4% men, p = 1.000). Significant differences in age groups were obtained for IF and IT, not for malignant IT. In case of administration of contrast agent, significant differences were only shown in IF (Table 5).

**Table 5:** Comparison of IF findings regarding age group and administration of contrast agent (IF: incidental finding, IT: incidentaloma, MIT: malignant incidentaloma).

	Age 18-64	Age>64	p-value	Contrast Agent	∅ Contrast Agent	p-value
**IF**	9.8%	32.6%	**<0.001**	26.6%	16.2%	**0.033**
**IT**	6.1%	19.9%	**<0.001**	14.1%	10.1%	0.321
**MIT**	0.2%	1.1%	0.070	0.5%	0.0%	1.000

When considering the modality, 129 CT scans and 24 MRI examinations showed IF. As a result, IF occurred in 31.1% of the 415 CT examinations and 4.9% of the 488 MRI examinations. There was a statistically significant relationship between modality and occurrence of IF (p < 0.001). Further analysis of IT revealed statistically significant association between modality and occurrence of IT (p < 0.001) with 18.8% of the CT scans and 3.3% of the MRI examinations. All four examinations with malignant IT were CT examinations. The MRI examinations did not reveal any malignant IT in all 488 patients. This resulted in a frequency of 1.0% for malignant IT in CT examinations with a statistically significant relationship between modality and occurrence of malignant IT (p = 0.031).

## 4 Discussion

Our results indicate that IF have a high prevalence in high resolution shoulder imaging and that they are more frequently found in CT scans compared to MRI examinations, presumably caused by the confined field of view as a limiting factor in MRI and the higher age in the CT population.

While IF occurred in 31.1% of the CT, IF were only detected in 4.9% of the MRI scans. Harmless IF occurred in 101 (11.2%) patients, IT in 94 (10.4%) and malignant IT in four (0.4%). Additionally, IF and IT prevalence are significantly higher in older patients. There was no significant correlation between prevalence of malignant IT findings and demographic data found.

In the literature, there exist limited evidence regarding the prevalence of IF in shoulder imaging. While there are only few studies investigating the prevalence of incidental shoulder enchondromas^11^ and chondral tumors^12^ in routine shoulder MRI, there is some evidence regarding IF in shoulder CT scans. In recent years, CT scans of the shoulder have increasingly been used not only for diagnostic purposes, but also for preoperative assessment of glenoid morphology, wear, version, inclination, and glenohumeral subluxation in anatomic total shoulder arthroplasties and reverse shoulder arthroplasties.^13^^,^^14^ Visualization of surrounding anatomical structures during shoulder scans helps identify to further IF.^15^

Previous studies investigating IF prevalence in shoulder imaging reported similar results to our findings. Chen et al. investigated the prevalence as well as the clinical impact of IF on preoperative 3D planning CT scans for total shoulder arthroplasty.^3^ With 39.3% IF findings, 11.4% potentially pathologic findings and 0.6% new cancer diagnosis, relative frequencies of the respective categories are close to our findings. Lopez et al. examined 302 patients to determine the prevalence and management of incidentally discovered pulmonary nodules in patients that received a preoperative CT scan of the shoulder for shoulder arthroplasty planning. At least one pulmonary nodule was found in 22.8% of the examined patients. In two patients (0.7%), the pulmonary nodule turned out to be malignant. These frequencies partly correspond to the results in the present work (22.8% pulmonary nodules vs. 18.8% IT and 0.7% malignant pulmonary nodules vs. 1.0% malignant IT). Although most pulmonary nodules are benign,^7^ the fact that lung cancer is a leading cause of mortality, and early detection of a tumor increases the chances of a favorable prognosis,^16^ illustrates the importance of exact and early detection of IF as well as sufficient workup strategies. Investigating these strategies, a recent study from Wengle et al. reported that all of their three patients with identified primary lung cancer were appropriately triaged and had repeat dedicated CT chest scans completed.^4^ However, seven patients proceeded with shoulder surgery without any pulmonary workup.^4^ This illustrates that beside the exact detection of IF, communication between medical disciplines is crucial to provide best workup and patient care.

Like shown in our own study, previous published studies reported that the frequency of IF occurrence varies between different modalities.^9^^,^^17^^,^^18^ A systematic review from Lumbreras et al. showed that the frequency of incidental findings is higher in studies involving CT technology.^9^ This may be caused related to the fact that CT is better than MRI in the display rate of bone and lung nodules and that CT and MRI indications differ. While CT indications include fractures or preoperative planning for shoulder arthroplasty, MRI is more likely used for e.g. the diagnosis of rotator cuff tears. Therefore, patient clientele varies between modalities.

Additionally, the prevalence of IFs not only varies with imaging modality, but is also influenced by patient age.^18^, ^19^, ^20^, ^21^, ^22^, ^23^, ^24^ In our own findings, the average age of patients with IF and IT was significantly higher compared to patients without IF and IT, presumably caused by the fact that older patients tend to have multimorbidity represented by multiple chronic diseases due to aging changes of organs.^25^ Only the age difference in malignant IT did not reach the level of significance, which may be attributed to the small number of patients with malignant IF (n = 4) in this cohort. Additionally, Staab et al. demonstrated that the number of IF increases with increasing age^26^ and also a statistical increase in the IF probability of 4.2% per year of life is described.^18^ In contrast, Lopez et al. found no significant age difference regarding incidental pulmonary nodules in shoulder arthroplasty preoperative CT scans.^15^ This was probably caused by the generally very high age of the entire study population of 70.7 years and therefore these findings cannot be transferred to younger age groups.^15^

In the literature, a strong association between higher weight and more frequent occurrence of IF and IT is reported.^27^ In contrast, we showed that the average weight of the patients with IF and IT was significantly lower compared to patients without IF and IT. A possible explanation for the opposite effect described in the present work is the distortion of the result due to the increased occurrence of IF in older patients and the simultaneous loss of body weight with age.^28^ Additionally, information about weight was only recorded for 47.2% of our total population group.

With an increasing number of imaging examinations in the future as well as the higher spatial resolution at which these examinations are carried out,^29^^,^^30^ the prevalence of IF will probably continue to increase over the next years.^20^^,^^31^ As the impact on patient morbidity and survival can be significant, the exact detection and initiation of appropriate therapy is of great clinical importance.^4^ Not only for patients, but also for the treating surgeons, identified IF on preoperative shoulder scans can be a source of uncertainty whether the originally planned procedure should be aborted, delayed or postponed and whether the workup of the IF should be given priority.^15^ Furthermore, Cochon et al. reported that the same examination can lead to different assessments of IF among different interpreting radiologists, leading to increased uncertainty in the appropriate clinical handling of IF.^32^

For the majority of IF, it can be stated that they usually do not represent true neoplasms or at least are not associated with clinical morbidity.^33^ Accordingly, the risk-benefit assessment of follow-up examinations for IF is not always easy, especially in asymptomatic patients.^19^ Thus, the detection of an IF that may ultimately prove to be harmless may lead to a cascade effect of additional diagnostic procedures and medical treatments, which may carry their own risks and harmful side effects for the patient.^34^ However, the detection of pathologies at an early stage enables more effective treatment^24^ and the greatest gain in health and lifespan is seen in the early detection of malignant processes.^8^ Ultimately, the focus should be on the patient and the limited time and resources in clinical practice should accordingly be spent on IFs that are most likely to be life-threatening.^1^ Requests for further examinations should fundamentally be based on the likelihood of an illness and not just on its possibility.^35^ Therefore, every clinician should fundamentally weigh the potential of any early detection of disease against possible disadvantages for the patient^9^ and medical history and symptoms should be included in the decision-making process.^36^

This study has some limitations that need to be mentioned. In this retrospective, descriptive study, the radiological findings were evaluated, but not the originally recorded examination material. Therefore, only IFs that were discovered and recorded by the radiologist could be evaluated. This leaves an uncontrollable risk of false-negative findings. Some studies whose examination field of view was too small to show relevant other parts of the body besides the shoulder were primarily excluded, but there may be instances in which the incidental finding is inherent to the structures of the shoulder itself (e.g., a chondroid lesion in the humeral head, an intramuscular lipoma within a rotator cuff muscle). Therefore, there could be some missed findings. A further limitation was the comparability of the examination volumes, especially of the CT examinations. Despite the same clinical question and the requested examination, volumes of different sizes were recorded and evaluated. Therefore, it can be assumed that studies with unusually small or large volumes could distort the determined prevalence. Furthermore, certain findings can be classified as either harmless or relevant depending on the patient's age.^9^ This reduced the comparability of the results with similar studies due to different age ranges. Therefore, a direct comparison and transfer of the results to other study populations was not fully possible. Information about height was only recorded for 40.5% and weight for only 47.2% of the total population group, due to lack of data in patient files. This leads to a reduced interpretation of these parameters. Additionally, the average age of the CT population was higher than the age of the MRI population (CT: 62.78 ± 18.71 years, MRI: 49.14 ± 14.91 years), which reduces the comparability between modalities.

## 5 Conclusion

IF have a high prevalence (16.9%), especially in CT examinations of the shoulder, which increases with age. With an increasing number of imaging examinations in the future and an aging society, the prevalence of IF will probably continue to increase over the next few years. The exact detection and initiation of appropriate therapy is therefore of great clinical importance, as early detection of life-threatening diseases enables more effective treatment and a potential gain in health and lifespan. Therefore, further evidence-based classification systems and guidelines should be developed to establish an effective and confident clinical management of IF.

## Funding statement

This research did not receive any specific grant from funding agencies in the public, commercial, or not-for-profit sectors.

## Patient's consent

Not applicable.

This retrospective study did not involve the use of human subjects.

## CRediT authorship contribution statement

**Mario Pasurka:** Investigation, Methodology, Project administration, Resources, Supervision, Validation, Visualization, Writing – original draft, Writing – review & editing, All authors must have made substantial contributions. **Adrian Statescu:** Formal analysis, Investigation, Methodology, Resources, Visualization, Writing – original draft, Writing – review & editing, All authors must have made substantial contributions. **Philipp von Knebel Doeberitz:** Resources, Software, Supervision, Validation, Visualization, Writing – review & editing, All authors must have made substantial contributions. **Joshua Kubach:** Methodology, Resources, Software, Supervision, Validation, Writing – review & editing, All authors must have made substantial contributions. **Franz Dally:** Data curation, Formal analysis, Supervision, Validation, Writing – review & editing, All authors must have made substantial contributions. **Sascha Gravius:** Data curation, Formal analysis, Supervision, Validation, Writing – review & editing, All authors must have made substantial contributions. **Marcel Betsch:** Data curation, Formal analysis, Supervision, Validation, Writing – review & editing, All authors must have made substantial contributions.

## Declaration of competing interest

None.
